# Identification of Oligopeptides in the Distillates from Various Rounds of Soy Sauce-Flavored Baijiu and Their Effect on the Ester–Acid–Alcohol Profile in Baijiu

**DOI:** 10.3390/foods14020287

**Published:** 2025-01-16

**Authors:** Qiang Wu, Shanlin Tian, Xu Zhang, Yunhao Zhao, Yougui Yu

**Affiliations:** 1 College of Food and Chemical Engineering, Shaoyang University, Shaoshui Road, Shaoyang 422000, China; palesilver2024@163.com (S.T.); zx17832519787@163.com (X.Z.); 13769193967@163.com (Y.Z.); yufly225@163.com (Y.Y.); 2 Hunan Province Key Laboratory of New Technology and Application of Ecological Baijiu Production, Shaoyang University, Shaoshui Road, Shaoyang 422000, China

**Keywords:** soy sauce aroma-flavored Baijiu, peptides, flavor substance, molecular docking

## Abstract

Endogenous peptides in Baijiu have primarily focused on finished liquor research, with limited attention given to the peptides in base liquor prior to blending. Liquid chromatography–tandem mass spectrometry (LC-MS) was employed to identify endogenous peptides in the distillates from the first to seventh rounds of soy sauce-flavored Baijiu. Two hundred and five oligopeptides were identified from these distillates, all of which had molecular weights below 1000 Da and were composed of amino acid residues associated with flavor (sweet, sour, and bitter) and biological activity. Furthermore, full-wavelength scanning, content determination of the main compounds, and molecular docking were performed to analyze these oligopeptides’ effect on the ester–acid–alcohol profile in Baijiu. This determination revealed a negative correlation between the peptide content and total ester content (r = −0.691), as well as the total acid content (r = −0.323), and a highly significant negative correlation with ethanol content (r = −0.916). Notably, the screened peptides (TRH, YHY, RQTQ, PLDLTSFVLHEAI, KHVS, LPQRHRMVYSLL, and NEWH) had specific interactions with the major flavor substances via hydrogen bonds, including esters (ethyl acetate, ethyl butanoate, ethyl hexanoate, and ethyl lactate), acids (acetate acid, butanoate acid, hexanoate acid, lactate acid), and alcohols (ethanol, 1-propanol, 1-butanol, and 1-hexanol). These findings elucidate the distribution and dynamic changes of endogenous peptides in the distillates from various rounds of soy sauce-flavored Baijiu, providing a theoretical foundation for further investigation into their interaction mechanisms associated with flavor compounds.

## 1. Introduction

Baijiu is one of China’s traditional distilled alcoholic beverages, produced from a mixture of grains and a fermentation agent prepared through a specialized process. Based on its aroma and flavor profiles, Baijiu is categorized into several distinct aroma types. Among these, the soy sauce-flavored Baijiu is notable for its unique production process. This variety is primarily crafted using sorghum as the main ingredient, while the saccharification and fermentation agents are derived from wheat, which serves as the culture medium during their preparation [1]. The production of Baijiu involves a highly intricate process, particularly for the soy sauce aroma style. This variety requires sorghum as the primary raw material and involves an initial fermentation and distillation step, followed by repeated cycles of fermentation and distillation, totaling eight distillations. The final product is derived by blending the liquors obtained from the last seven distillations in specific proportions. These seven distillation cycles are referred to as the first through seventh rounds of liquor [2].

In recent years, research on Baijiu has primarily focused on characterizing bacterial communities and conducting preliminary investigations into the formation mechanisms of flavor compounds [3], as well as identifying volatile components. However, relatively little attention has been given to non-volatile substances. The primary non-volatile compounds reported in Baijiu include amino acids, reducing sugars, higher fatty acids, polyols, minerals, vitamins, and peptides [4]. Peptides extracted from Baijiu exhibit a range of metabolic and physiological regulatory functions, including antimicrobial, anticancer, antioxidant, and antihypertensive activities [5]. A novel tetrapeptide has been identified, which exerts vasodilatory effects in angiotensin I-stimulated endothelial cells through the eNOS and END1 pathways [6]. A distinct advantage of these peptides is their superior digestibility and bioavailability compared to other flavor-related compounds.

In addition, endogenous oligopeptides in Baijiu can interact with aroma or odor components, resulting in changes to the volatilization properties of the latter and maintaining aroma, changing flavor type, and covering odor [7,8,9]. The tetrapeptide Asp-Arg-Ala-Arg (DRAR), identified in sesame-flavored Baijiu, was found to inhibit the volatilization of aromatic compounds at concentrations ranging from 10 to 1000 μg/L, with inhibition levels ranging from 0.09% to 39.2%. Notably, DRAR exhibited stronger inhibitory effects on esters and alcohols [7]. The peptide Ala-Lis-Arg-Ala (AKRA) in sesame-flavored Baijiu interacts with p-cresol via hydrogen bonding to form an AKRA-p-cresol complex, thereby reducing the headspace concentration of phenolic compounds [8]. It has been found that the cyclic peptide digitonin in strongly flavored Chinese Baijiu selectively influences the volatilization of aromatic compounds, effectively inhibiting the volatilization of phenolic compounds at a concentration of 160 μg/L, with an inhibition rate of 36–48% (*p* < 0.05) [9].

However, research on endogenous peptides in soy sauce-flavored Baijiu remains limited. Most studies have focused primarily on the investigation of endogenous peptides in finished liquor, with less attention given to the entire production process. Furthermore, no studies have analyzed the changes in peptides derived from different distillation rounds. As a result, the mechanisms underlying their formation during the distillation process remain unclear, hindering efforts to optimize peptide concentrations in subsequent blending stages. This study primarily focuses on the identification of endogenous peptides in different distillation rounds of soy sauce-flavored Baijiu, and it further analyzes the changes in the levels of peptides, esters, acids, and alcohols and the correlations between these components. The findings provide valuable insights into the variations in the content of peptides, esters, acids, and alcohols in soy sauce-flavored Baijiu. These results offer a useful reference for further studies on the production mechanism of endogenous peptides involved in Baijiu fermentation.

## 2. Materials and Methods

### 2.1. Chemicals and Reagents

Methanol and acetonitrile (99% purity) were purchased from Aladdin Co., Ltd. (Shanghai, China). All standards, including lactic acid, propionic acid, caproic acid, ethanol, methanol, amyl alcohol, n-propanol, ethyl lactate, ethyl acetate, and ethyl formate, were obtained from Sigma-Aldrich (St. Louis, MO, USA). All other chemicals and reagents used in this study were of analytical grade and were purchased from Sinopharm Chemical Reagent Co., Ltd. (Beijing, China).

### 2.2. Preparation of Basic Liquor from Different Distillation Rounds of Soy Sauce-Flavored Baijiu

Liquors were collected during seven distillation rounds from Xiangjiao Brewing Ltd. (Shaoyang, China) in accordance with the process for soy sauce-flavored Baijiu. Each round of distillation lasted 50 min, and the liquors were sampled with distillations from 5 to 20 min and had an ethanol content ranging from 52 to 55% (*v*/*v*) and a yield of 85% (*v*/*v*). Each sample was analyzed in triplicate, and the collected liquors were sealed and stored at 4 °C until further analysis.

As described by Zhang et al. [9], a 100 mL liquor sample was freeze-dried. The resulting freeze-dried powder was then processed using a 10 kDa Millipore ultrafiltration tube (15 mL, Millipore, Billerica, MA, USA) according to the manufacturer’s instructions. These filtrates were demineralized using an Empore solid-phase extraction column C18 (7 mm I.D., 3 mL, Sigma-Aldrich, St. Louis, MO, USA), which was pre-equilibrated using 40 mL of 10% acetonitrile under normal pressure. After washing with 5 mL of 10% acetonitrile, the peptide fractions were eluted with 5 mL of 70% acetonitrile. The eluents were evaporated in a vacuum concentration system to remove acetonitrile. The peptide mixture obtained was subsequently freeze-dried using a vacuum centrifugation concentrator (Thermo Fisher Scientific, Waltham, MA, USA).

### 2.3. Separation of Peptides from Basic Liquor of Soy Sauce-Flavored Baijiu

As described by Zhang et al. [9], the separation of peptides was carried out using an Easy nLC HPLC system (ThermoFisher Scientific, Waltham, MA, USA), which employs a nanoliter flow rate. A 40 µL sample of the freeze-dried, 0.1% TFA redissolved soy sauce-flavored Baijiu was applied to a Zorbax 300SBC18 peptide trap (15 cm length, 75 µm inner diameter, Agilent Technologies, Wilmington, DE, USA) via an automatic sampler. The peptides were separated using a C18 column (75 µm × 150 mm, Column Technology Inc., Fremont, CA, USA) at a flow rate of 250 nL/min. The column was equilibrated with 95% liquid phase A. The gradient for the mobile phase was as follows: from 0 to 50 min, the B phase increased linearly from 4% to 50%; from 50 to 54 min, the B phase increased linearly from 50% to 100% from 54 to 60 min, and the B phase was maintained at 100%. Phase A was a 0.1% formic acid aqueous solution, and phase B was a 0.1% formic acid acetonitrile aqueous solution (84% acetonitrile).

### 2.4. Identification of Peptides from Basic Liquor of Soy Sauce-Flavored Baijiu

The peptide fraction was identified using mass spectrometry with a Q Exactive mass spectrometer (Thermo Fisher Scientific, Waltham, MA, USA) [10]. The analysis duration was 60 min. The detection method employed a positive ion mode, with a parent ion scanning range of 300–1800 *m*/*z*. The primary mass spectrum resolution was set to 70,000 (at *m*/*z* 200), with an automatic gain control (AGC) target of 1e6. The maximum ion trap (IT) value was set to 10 ms, and only one scan range was performed. Dynamic exclusion was set to 20.0 s. The mass-to-charge ratios of peptides and their fragments were collected in the following manner: 10 fragment spectra (MS2 scans) were recorded after each full scan. The MS2 activation type was set to high-energy collision dissociation (HCD), with an isolation window of 1.6 *m*/*z*. The secondary mass spectrometry resolution was 17,500 (at *m*/*z* 200), with one microscan. The maximum IT value was 60 ms, and the normalized collision energy was set to 27 eV. The underfill ratio was maintained at 0.1%.

### 2.5. Full-Wavelength Scanning

As described by Zhang et al. [11], full-wavelength scanning was performed using an ultraviolet/visible spectrophotometer (UV2700; Shimadzu, Nakagyo-ku, Kyoto, Japan). Lactic acid, propionic acid, caproic acid, ethanol, methanol, amyl alcohol, n-propanol, ethyl lactate, ethyl acetate, and ethyl formate were dissolved in a 50% *v*/*v* ethanol/aqueous solution at a concentration of 10 µL/mL. Both the standard solutions and samples were scanned across the full-wavelength range, with a scanning range from 185 to 1400 nm. A 750 µL sample was aspirated into a 5 mm diameter test tube. After full-wavelength scanning, the maximum absorption values of characteristic peaks corresponding to acids, esters, and peptides (185–200 nm, 200–220 nm, 220–250 nm, and 250–300 nm) were analyzed.

### 2.6. Determination of the Main Chemical Indices of the Collected Liquor

The peptide concentration in the collected liquors was determined using the Coomassie brilliant blue staining method [12]. Briefly, the sample was concentrated using a rotary evaporator (R100, Buchi, Shanghai, China), and 500 µL of the liquor concentrate was added to 2.5 mL of Coomassie Brilliant Blue solution. The mixture was shaken well and allowed to stand for 5 min, and the absorbance was measured at 595 nm using an ultraviolet/visible spectrophotometer (UV2700; Shimadzu, Nakagyo-ku, Kyoto, Japan) to calculate the peptide concentration.

The contents of total acid and total ester were determined by acid–base titration [13,14]. A 50 mL aliquot of the liquor was placed in a conical flask, and two drops of phenolphthalein indicator (10 g/L) were added. The acid in the sample was neutralized using a 0.1 mol/L sodium hydroxide standard solution. Titration was completed when the solution turned pink, and the volume of sodium hydroxide required was recorded to calculate the total acid content.

For the determination of the total ester content, 25 mL of excess sodium hydroxide was added to the sample, mixed thoroughly, and the mixture was refluxed in a boiling water bath. After cooling for 30 min, the sample was titrated with 0.1 mol/L sulfuric acid standard solution. The endpoint was reached when the solution changed from faint red to colorless. The volume of sulfuric acid required for titration was recorded, and the total ester content was calculated.

The above determinations were performed in triplicate, and the average values were used. Ethanol content in the different rounds of soy sauce-flavored Baijiu was measured using an electronic liquid densimeter (BHOM-SJ04, Shijiazhuang Baiheng General Instrument Manufacturing Ltd., Shijiazhuang, China).

### 2.7. Molecular Docking

The 3D structure of the peptides and the main flavor substances were drawn using ChemBioDraw 14.0 (OriginLab Co., Ltd. CambridgeSoft, Cambridge, MA, USA) and converted into a pdb file. AutoDock Tools, AutoGrid, and AutoDock modules in the AutoDock 4.2.6 software were used to simulate molecular docking between the peptides and the main flavor substances [11].

### 2.8. Statistical Analysis

All data were analyzed using Statistical Product and Service Solutions (SPSS, version 21.0, IBM, Armonk, NY, USA). The least significant difference (LSD) range test was applied to determine significant differences (*p* < 0.05). Spearman’s rank correlation coefficient was used for correlation analysis, and SPSS software was employed to examine the relationship between peptide accumulation and the volatilization of main flavor substances across the different distillation rounds of liquor.

## 3. Results and Discussion

### 3.1. Structural Characterization of Peptides in the Distillates from Various Rounds Soy Sauce-Flavored Baijiu

The amino acid sequences of peptides in the distillates from the first to seventh rounds of soy sauce-flavored Baijiu were identified using LC-MS in combination with de novo sequencing. A total of 46 peptides were identified in the first round of soy sauce-flavored Baijiu, with the identification results presented in Table 1. In the second round, 35 peptides were identified (Table 2), while 40 peptides were identified in the third round (Table 3). In the fourth round, 19 peptides were detected (Table 4), and 18 peptides were identified in the fifth round (Table 5). The sixth round contained 19 peptides (Table 6), and the seventh round contained 28 peptides (Table 7).

Based on the confidence score, peak area, and retention time, representative peptide structures with high confidence, large peak areas, and short retention times were selected. The identified peptides from the first to seventh rounds include the following: Thr-Arg-His (TRH, *m*/*z*: 412.22), Tyr-His-Tyr (YHY, *m*/*z*: 481.20), Arg-Gln-Thr-Gln (RQTQ, *m*/*z*: 531.28), Pro-Leu-Asp-Leu-Thr-Ser-Phe-Val-Leu-His-Glu-Ala-Ile (PLDLTSFVLHEAI, *m*/*z*: 1453.78), Lys-His-Val-Ser (KHVS, *m*/*z*: 469.27), Leu-Pro-Gln-Arg-His-Arg-Met-Val-Tyr-Ser-Leu-Leu (LPQRHRMVYSLL, *m*/*z*: 1511.84), and Asn-Glu-Trp-His (NEWH, *m*/*z*: 584.23), which, to our best knowledge, has not been reported. These peptides were different from the oligopeptides found in other flavored Baijiu, which contain some amino acids related to biological activity, such as Gln-Gly-Val-Pro (QGVP) from sesame-flavored Baijiu [6] and DRAR [7] and AKRA [8] in sesame-flavored Baijiu. It has been reported that hydrophobic amino acid residues frequently appear in oligopeptides derived from Baijiu related to its flavor. The total ion chromatograms for the first to seventh rounds and their corresponding mass spectra were shown in Figure S1. Moreover, as shown in Table S1, KHVS was detected in both the first and second rounds of soy sauce-flavored Baijiu, while NEWH was detected in both the first and third rounds.

**Figure 1:**
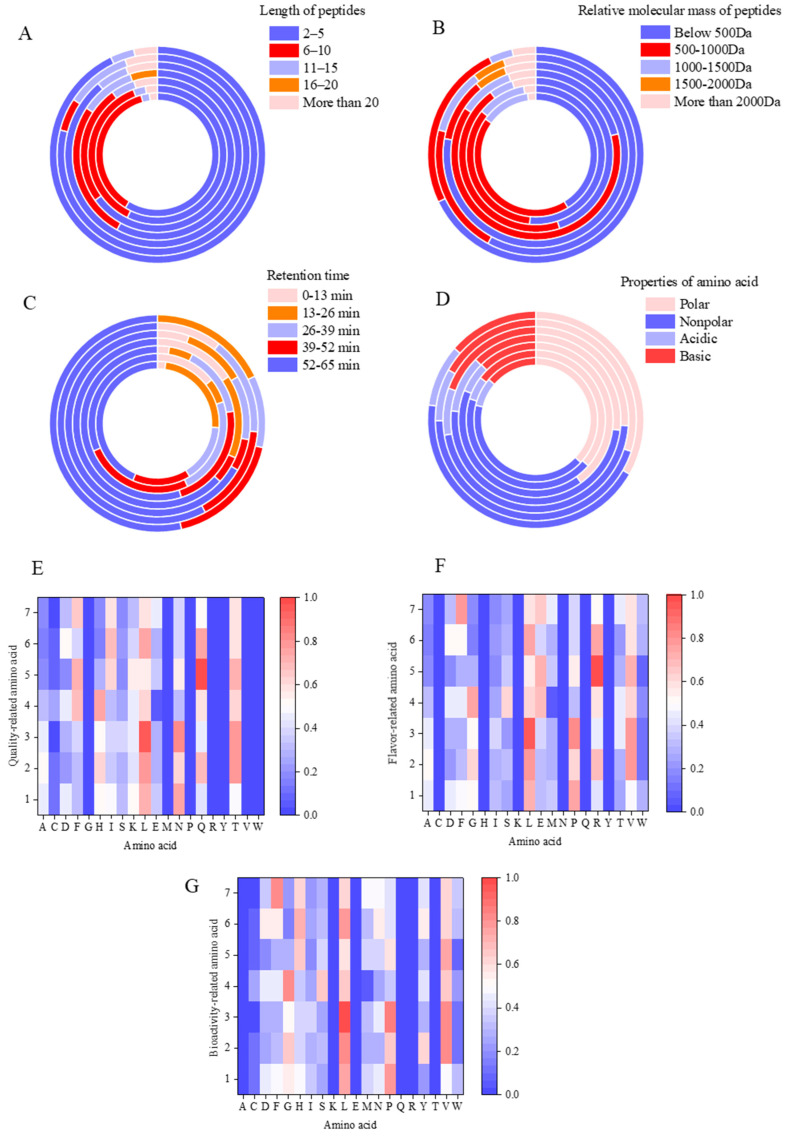
Statistical analysis of the amino acid residues that occurred in peptides of the liquors from different rounds. (**A**) Distribution diagram of the length of the peptides. It was calculated as the proportion in the totality of amino acid residues of all peptides. The circles from the inside to the outside represent the one to seven rounds of liquor. (**B**) Distribution diagram of the molecular weight of the peptides. It was calculated as the proportion in the totality of amino acid residues of all peptides. The circles from the inside to the outside represent the one to seven rounds of liquor. (**C**) Distribution diagram of the retention time of the peptides. It was calculated as the proportion in the totality of amino acid residues of all peptides. The circles from the inside to the outside represent the one to seven rounds of liquor. (**D**) Distribution diagram of the amino acid properties of the peptides. It was calculated as the proportion in the totality of amino acid residues of all peptides. The circles from the inside to the outside represent the one to seven rounds of liquor. (**E**) Distribution diagram of amino acids influenced the Baijiu quality of the liquors. It was calculated as the proportion of the totality of amino acid residues of all peptides. (**F**) Distribution diagram of the flavor-related amino acid of the liquors. It was calculated as the proportion in the totality of amino acid residues of all peptides. (**G**) Distribution diagram of the bioactive amino acid of the liquors. It was calculated as the proportion in the totality of amino acid residues of all peptides.

Based on the amino acid sequences of peptides collected from different rounds, it was observed that oligopeptides predominated in all rounds of soy sauce-flavored Baijiu. Peptides isolated from the first to fourth rounds primarily consisted of 2 to 10 amino acids, while peptides from the fifth to seventh rounds were mostly composed of 2 to 5 amino acids (Figure 1A). The molecular weights of peptides from the first to third rounds generally fell below 1000 Da, while those from the fourth round ranged between 500 and 1000 Da. In contrast, peptides from the sixth and seventh rounds mostly had molecular weights below 500 Da (Figure 1B). The retention times of peptides across all rounds were mainly between 52 and 65 min, with an increasing number of peptides detected as the analysis progressed (Figure 1C). Furthermore, there was a higher prevalence of polar and basic amino acids in the peptides from the first to seventh rounds (Figure 1D).

Specific amino acids, such as Ala, Gly, Leu, Glu, Lys, Pro, Arg, and Val, have been reported to positively influence the quality of liquor [7,8]. These amino acids, including Ala, Gly, Leu, Glu, Pro, Arg, Thr, and Val, are also associated with various taste profiles, such as sweet, sour, and bitter [15]. Additionally, key amino acids with known biological activity, including Ala, Leu, Glu, Pro, Arg, Lys, Thr, and Val, have been identified in previous studies [16,17,18]. As shown in Figure 1E, among the amino acids influencing liquor quality, Lys, Leu, Pro, and Gly were found in relatively high proportions in the first round of soy sauce-flavored Baijiu. In the second round, Gly, Arg, Leu, Ala, Val, and Pro were more prevalent. Gly, Leu, Pro, and Val were commonly found in the third round, while Gly, Arg, Leu, Glu, and Val were more prominent in the fourth round. In the fifth round, His, Lys, Leu, Glu, Val, Pro, and Arg were more common. The sixth round showed a higher presence of His, Leu, Asp, Val, and Arg, while His, Leu, Glu, Val, and Arg were more prevalent in the seventh round.

Umami is a highly desirable taste, primarily induced via glutamic acid. In Baijiu, umami substances are mainly derived from amino acids, which are broken down from proteins and contribute to a pleasant, sweet taste. Increasing the amino acid content in liquor can enhance its quality. The type, content, and proportion of amino acids play a significant role in shaping the flavor of liquor. While many amino acids impart a primarily umami flavor, some also contribute bitter or salty notes. In Baijiu, these amino acids combine with other trace compounds to evolve, forming a distinctive, light, and elegant flavor profile characterized by a clean and delicate mouthfeel. This interaction enhances the overall harmony and fullness of the liquor’s body. Among the amino acids, Ala, Gly, Asp, Pro, Thr, and Ser are associated with sweetness, while Leu, Val, Pro, Arg, Try, Met, and Phe are linked to bitterness. As shown in Figure 1F, among the flavor-related amino acids, Leu, Pro, and Gly were found in relatively high proportions in the first round of soy sauce-flavored Baijiu. In the second round, Gly, Arg, Leu, Ala, Val, and Pro were more prevalent. In the third round, Gly, Leu, Pro, Thr, and Val dominated. In the fourth round, Gly, Ser, Leu, Glu, Arg, Thr, and Val were more common. The fifth round was characterized by a higher presence of Leu, Glu, Val, Pro, and Arg. In the sixth round, Ala, Leu, Asp, Val, Arg, Try, and Phe were more prominent. Finally, in the seventh round, Met, Ala, Leu, Glu, Val, Arg, Thr, Try, and Phe were more abundant. Additionally, the first two rounds of liquor exhibited a sour and astringent taste, while the third round began to show a slight balance of sourness and sweetness. The sixth round was characterized by a subtle sweetness and bitterness, and the seventh round displayed a mild sour and bitter profile. The experimental results also revealed significant differences in the flavor amino acid composition across the first to seventh rounds of soy sauce-flavored Baijiu. This highlights the importance of exploring the different endogenous peptides that contribute to the distinct flavor profile of the liquor.

As shown in Figure 1G, among the amino acids reported to have biological activity, Leu, Proline, Gly, and Try make up a relatively large proportion in the first round of soy sauce-flavored Baijiu. In the second round, Gly, Tyr, Leu, Ala, Val, and Pro were more prevalent. In the third round, Gly, Leu, Pro, and Val were more commonly found. The fourth round was characterized by a higher presence of Gly, Ser, Leu, Thr, and Val. In the fifth round, Leu, His, Val, Pro, and Arg were more abundant. The sixth round showed a greater proportion of His, Tyr, Ala, Leu, Asp, Val, Arg, Try, and Phe. Finally, in the seventh round, Met, Ala, Leu, His, Val, Try, and Phe were more prevalent. These findings indicated that there was a higher diversity of amino acids with biological activity in the different rounds of soy sauce-flavored Baijiu, with leucine being particularly abundant across all rounds.

### 3.2. Correlation Analysis of Peptides Derived from Different Distillation Rounds and Main Components

**Figure 2:**
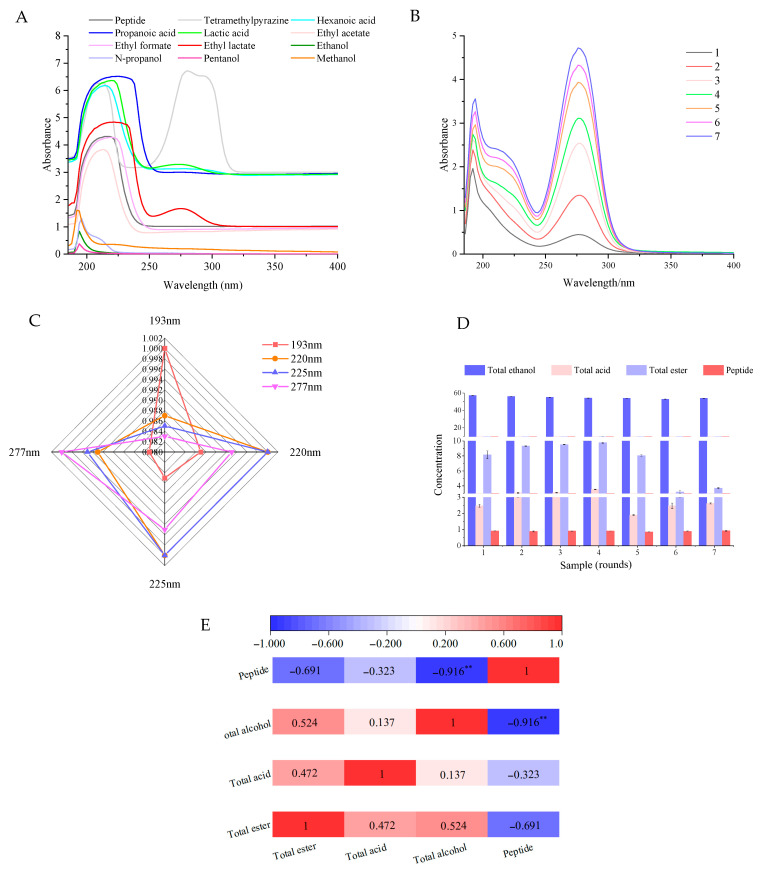
Correlation analysis between the peptides and the main flavor substances in the liquors collected at different rounds. (**A**) Spectra of the standard substances involved in Baijiu. (**B**) Spectra of the liquors from different rounds. (**C**) Correlation analysis among the characteristic peaks based on the maximum absorption value. (**D**) Content determination of total acid, total ester, total alcohol, and the peptide. (**E**) Correlation analysis between the content of the peptide and total acid, total ester, or total alcohol. Red represents positive, and blue represents negative. ** *p* ≤ 0.01.

The main components in liquor are alcohols, esters, and acids [19]. Previous studies have performed full-wavelength scans of key flavor substances, such as alcohols, acids, and esters. As shown in Figure 2A, alcohols (ethanol, methanol, n-butanol, and n-propanol) exhibit characteristic peaks within the 185–200 nm range, while acids and 2,3,5,6-tetramethylpyrazine display distinct peaks at 200–220 nm, 220–250 nm, and 250–300 nm, respectively [20,21,22]. Peptide substances are known to exhibit characteristic peaks in the 200–220 nm range [23], while esters typically show peaks between 250 and 300 nm.

Full-wavelength scanning of the different rounds of soy sauce-flavored Baijiu revealed significant differences in the absorption profiles across the 185–200 nm, 200–220 nm, 220–250 nm, and 250–300 nm regions (Figure 2B). The correlation analysis of the peak intensities across these four wavelength segments for liquor samples from different rounds was presented in Figure 2C. The maximum absorption values at 193 nm and 220 nm exhibited a strong positive correlation (r = 0.987), indicating a close relationship between these wavelengths. Similarly, absorption peaks at 225 nm (r = 0.985) and 277 nm (r = 0.983) showed a significant positive correlation. Additionally, the absorption peak at 225 nm was positively correlated with those at 220 nm (r = 1) and 277 nm (r = 0.995). A strong correlation was also observed between the absorption values at 220 nm and 277 nm (r = 0.993).

The contents of peptide, total acid, total ester, and ethanol in different rounds of soy sauce-flavored Baijiu were tested, and their correlations were analyzed. As shown in Figure 2D, the total ester content in the first to fourth rounds of soy sauce-flavored Baijiu increased from 8.16 g/L to 9.77 g/L and then decreased to 3.19 g/L. The seventh round showed a slight increase to 3.70 g/L. The total acid content also increased from 2.47 g/L to 3.53 g/L in the first to fourth rounds and then decreased to 1.90 g/L in the fifth round, and increased to 2.47 g/L in the sixth and 2.62 g/L in the seventh rounds. The ethanol content showed an overall downward trend from 57.1% (*v*/*v*) to 52.9% (*v*/*v*), rising to 53.9% (*v*/*v*) in the seventh round. The peptide concentration in the different rounds of soy sauce-flavored Baijiu showed fluctuating changes. It first decreased in the first to seventh rounds, then increased, then decreased again, and finally increased in the seventh round to reach the maximum value of 0.93 mg/mL. In general, the peptide content in different rounds of soy sauce-flavored Baijiu did not differ significantly, but the content was highest in the first and seventh rounds.

As shown in Figure 2E, the contents of total ester and total acid (r = 0.472) and alcohol (r = 0.524) were positively correlated with each other, while the contents of total ester and peptide were negatively correlated (r = −0.691). A positive correlation was observed between total acid content and alcohol content (r = 0.137), whereas a negative correlation was found between total acid content and peptide content (r = −0.323). There was a significant negative correlation between peptide content and ethanol content (r = −0.916, *p* < 0.01). These results suggest that the correlation between peptide content and alcohol content is the strongest among the various components in the different rounds of soy sauce-flavored Baijiu. This may be attributed to the large differences in acidity, temperature, and microbial content in the fermented grains during different rounds of fermentation [22,23], which in turn affect various indices in distilled soy sauce-flavored Baijiu.

### 3.3. Interaction of Peptides Derived from Different Distillation Rounds and Main Components

**Figure 3:**
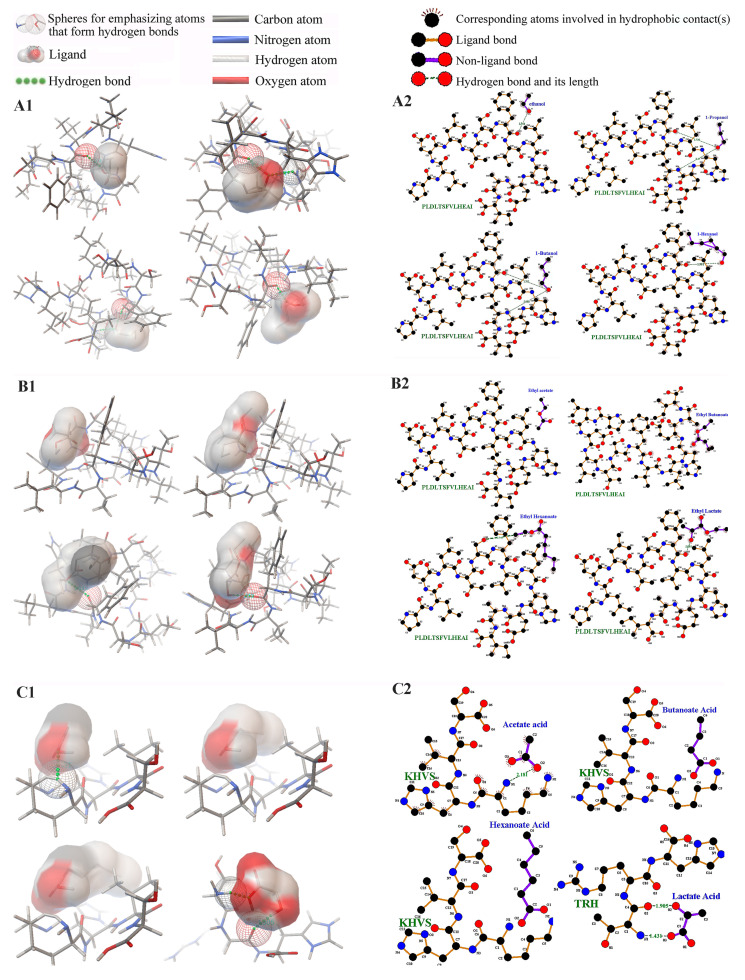
Molecular docking of the peptides with a flavor substance. (**A1**) PLDLTSFVLHEAI and the alcohols. (**B1**) PLDLTSFVLHEAI and the ester substances. (**C1**) KHVS or TRH and the acid substances. The interaction force model of the peptide and flavor substance molecules. (**A2**) PLDLTSFVLHEAI and the alcohols. (**B2**) PLDLTSFVLHEAI and the ester substances. (**C2**) KHVS or TRH and the acid substances.

Seven representative peptides were selected as receptors, while flavor substances were used as ligands for a one-to-one molecular docking analysis. The results revealed that the torsion energy of each peptide remained constant when interacting with the same flavor substance (Figure 3 and Table S2). Based on these observations, the maximum and minimum binding energy values were highlighted for comparison. Among the findings, the peptide PLDLTSFVLHEAI exhibited the highest binding energy with alcohols and esters, while KHVS showed the strongest binding with acetic acid, butyric acid, and caproic acid, and TRH demonstrated the highest binding energy with lactic acid. The binding energy of alcohols with PLDLTSFVLHEAI ranged from −1.89 to −2.30 kcal/mol, while esters ranged from −2.31 to −2.65 kcal/mol. For acids, binding energy values ranged from −3.12 to −3.19 kcal/mol with KHVS or TRH.

The van der Waals, hydrogen bond, and desolvation energy (Vdw-Hb-Desolv energy) contributed favorably to binding (negative values), while electrostatic energy was generally conducive to binding but occasionally unfavorable (positive values). Torsion energy, however, consistently hindered binding. For alcohols and esters binding with PLDLTSFVLHEAI, the Vdw-Hb-Desolv energy ranged from −2.16 to −3.74 kcal/mol and −2.94 to −4.24 kcal/mol, respectively. For acids binding with KHVS or TRH, the Vdw-Hb-Desolv energy ranged from −0.97 to −2.34 kcal/mol, while the electrostatic energy ranged from −2.01 to −2.44 kcal/mol.

The electrostatic energy values for alcohols and esters binding with peptides were relatively small, ranging from −0.03 to −0.09 kcal/mol for alcohols and −0.02 to 0.04 kcal/mol for esters. Torsion energy tended to increase with the carbon chain length; for example, the torsion energy of butyric acid (0.89 kcal/mol) was higher than that of acetic acid (0.30 kcal/mol), and the torsion energy of ethyl butyrate (1.19 kcal/mol) exceeded that of butyric acid.

Interestingly, not all hydrogen bonds correlated with high Vdw-Hb-Desolv energy values. For instance, while PLDLTSFVLHEAI exhibited the highest Vdw-Hb-Desolv energy with ethyl butyrate, no hydrogen bond was formed in this case. On the other hand, PLDLTSFVLHEAI demonstrated the lowest binding energy with ethanol, and the binding energies of KHVS and TRH with acids were higher than with ethanol. This suggests that PLDLTSFVLHEAI may have a positive impact on flavor in high-alcohol liquors, while KHVS or TRH could enhance flavor in low-alcohol liquors.

The structural analysis further revealed specific hydrogen bond formations: Ala(H) or Phe(O) of PLDLTSFVLHEAI formed hydrogen bonds with alcohols, with bond lengths ranging from 1.736 Å to 2.088 Å. Similarly, Ser(O) and Phe(O) of PLDLTSFVLHEAI formed hydrogen bonds with ethyl butyrate and ethyl lactate, with bond lengths of 2.945 Å and 2.092 Å, respectively. Lys(H) of KHVS formed a hydrogen bond with ethyl acetate, with a bond length of 2.181 Å. Furthermore, Thr(H) or Arg(O) of TRH formed hydrogen bonds with ethyl lactate, with bond lengths of 1.431 Å and 1.905 Å, respectively.

These findings highlight the nuanced interactions between peptides and flavor substances, offering valuable insights into their roles in enhancing the sensory profiles of liquor.

## 4. Conclusions

Two thousand and five peptides were discovered in the distillates from seven rounds of soy sauce-flavored Baijiu identified via LC-MS. The distillate from the first round was rich in oligopeptides, the amino acid length of which mainly ranged from 2 to 10 amino acids. These identified peptides had potential benefits on Baijiu quality, potentially influencing acidity, bitterness, and sweetness, and they even exhibited biological activity. Furthermore, the peptide content was negatively correlated with the ester–acid–alcohol content. The molecular docking results showed that peptides such as TRH, YHY, RQTQ, PLDLTSFVLHEAI, KHVS, LPQRHRMVYSLL, and NEWH bound with the esters (ethyl acetate, ethyl butanoate, ethyl hexanoate, and ethyl lactate), the acids (acetate acid, butanoate acid, hexanoate acid, and lactate acid), and the alcohols (ethanol, 1-propanol, 1-butanol, and 1-hexanol) via hydrogen bonds. This study revealed the changes to endogenous peptides in different distillation rounds of soy sauce-flavored Baijiu, and the study explored these changes’ correlation with the ester–acid–alcohol profile and interaction with the main flavor compounds, which provided a theoretical basis for liquor enterprises to improve the distillation process in order to increase the peptide content, thereby improving the quality of Baijiu.

## Figures and Tables

**Table 1:** Composition of peptide in the first round of soy sauce-flavored Baijiu.

Sample	Sequence	Length	Mass	Sequence	Length	Mass
The first round	SDAE	4	420.14924	MVTETLNFEHSNIQVKDFIMG	21	2452.1767
	TRLF	4	535.31183	ALDSSHERP	9	1010.4781
	WAK	3	403.22195	IPDLQVETNQGKI	13	1453.7777
	NVLH	4	481.26488	IIPAECRWR	9	1142.6019
	WVP	3	400.21106	FDHGFAEQ	8	949.393
	NMLP	4	473.2308	LQMARGGI	8	844.45891
	YPH	3	415.18557	AGDDKKNRD	9	1017.4839
	LHR	3	424.25465	IADELFFMP	9	1081.5154
	KHVS	4	469.26488	LDMYLNLY	8	1043.4998
	NYFV	4	541.25365	PSRDLMAY	8	951.4484
	FCF	3	415.15658	NCAKKGDP	8	831.39089
	MDF	3	411.14641	KHAPTVQS	8	866.46102
	PPNF	4	473.22743	PPLPTKVG	8	807.48544
	LFNP	4	489.25873	RILMVGPG	8	841.48439
	WSW	3	477.20122	HHHCAAAR	8	901.40894
	WRD	3	475.21793	LHVKGLQK	8	921.57599
	RHE	3	440.21318	LLGKLGGL	8	769.50617
	NEWH	4	584.23431	PKQKKLPI	8	950.62769
	QPAY	4	477.22235	VVNIPVVG	8	795.48544
	KHGE	4	469.2285	PPFTG	5	517.25365
	TRH	3	412.21827	PLFVN	5	588.32715
	QPMQ	4	502.22097	GWFWW	5	780.33838
	MKY	3	440.20934	RMND	4	534.22203

**Table 2:** Identification of peptides in the second round of soy sauce-flavored Baijiu.

Sample	Sequence	Length	Mass	Sequence	Length	Mass
The second round	RKF	3	449.27505	LPLEELPAIMEAI	13	1437.7789
	TRLF	4	535.31183	DLYANTVLSGGTTMYPGIADR	21	2214.0627
	RRQ	3	458.27136	ICYLQILR	8	1020.579
	KHVS	4	469.26488	NAWGALVQ	8	857.43955
	WRD	3	475.21793	VVSPLSSL	8	800.46437
	VHY	3	417.20122	GVRWFRYL	8	1095.5978
	PPNF	4	473.22743	LLHCQGHP	8	903.43851
	TREY	4	567.26528	VGHKMIVF	8	929.51569
	LFNP	4	489.25873	KGIFGVGS	8	763.42284
	VMY	3	411.18279	NKKAVLLV	8	883.58549
	KHGE	4	469.2285	PLHRPPPL	8	925.54977
	RNN	3	402.19753	RRVERRRA	8	1097.6642
	MDF	3	411.14641	VVPKAGSV	8	755.45414
	YHY	3	481.19613	YYAGPRAA	8	867.4239
	HDY	3	433.15975	PKGLPVVG	8	765.47487
	TEY	3	411.16416	VCGY	4	440.17296
	EDM	3	393.12059	TKAS	4	405.22235
	MKY	3	440.20934			

**Table 3:** Identification of peptides in the third round of soy sauce-flavored Baijiu.

Sample	Sequence	Length	Mass	Sequence	Length	Mass
The third round	RKF	3	449.27505	MVTETLNFEHSNIQVKDFIMG	21	2452.1767
	QKL	3	387.24817	LPLEELPAIMEAI	13	1437.7789
	RRQ	3	458.27136	PLDLTSFVLHEAI	13	1453.7817
	TRLF	4	535.31183	DLYANTVLSGGTTMYPGIADR	21	2214.0627
	WAK	3	403.22195	PLHMHGGN	8	861.39156
	HFD	3	417.16483	RQKAMALL	8	929.54806
	RHV	3	410.239	VAPKIIGG	8	753.47487
	NYFV	4	541.25365	YVVLTKLI	8	947.60555
	QPAY	4	477.22235	LLGKLGGL	8	769.50617
	VEE	3	375.16416	PKLPPSLG	8	807.48544
	WLSD	4	519.23291	VANAPLPL	8	793.46979
	VRQ	3	401.23867	VLDLPKVV	8	881.5586
	RNN	3	402.19753	VVGINPPV	8	793.46979
	RCY	3	440.18419	VVNIPVVG	8	795.48544
	NEWH	4	584.23431	PPFTG	5	517.25365
	TRH	3	412.21827	PPTSH	5	537.25471
	RNK	3	416.24957	SHDPP	5	551.23398
	TEY	3	411.16416	RQTQ	4	531.27651
	MEY	3	441.15697	MKVP	4	473.26719
	QKHA	4	482.26013	NMLP	4	473.2308

**Table 4:** Identification of peptides in the fourth round of soy sauce-flavored Baijiu.

Sample	Sequence	Length	Mass	Sequence	Length	Mass
The fourth round	EDRP	4	515.23398	HDSLFFVNEIK	11	1347.6823
	VFFSD	5	613.27478	PLDLTSFVLHEAI	13	1453.7817
	EELL	4	502.26388	TINELGGKYSGMDRYEARK	19	2187.0743
	VNKSD	5	561.27584	RFTAGGSS	8	781.37187
	TRK	3	403.25432	GVCYGFQW	8	958.40072
	RGEY	4	523.23906	VWCGPCRA	8	890.38911
	TREY	4	567.26528	YNGSVAGC	8	769.30649
	KHGE	4	469.2285	VRLITTLL	8	927.6117
	KHVS	4	469.26488	SDPHE	5	583.2238
	WVP	3	400.21106			

**Table 5:** Identification of peptides in the fifth round of soy sauce-flavored Baijiu.

Sample	Sequence	Length	Mass	Sequence	Length	Mass
The fifth round	RRQ	3	458.27136	MVTETLNFEHSNIQVKDFIMG	21	2452.1767
	RKF	3	449.27505	LPQRHRMVYSLL	12	1511.8395
	RQP	3	399.22302	TKAS	4	405.22235
	WVP	3	400.21106	NVLH	4	481.26488
	RNK	3	416.24957	RGEY	4	523.23906
	VEE	3	375.16416	PPVRC	5	570.2948
	LHR	3	424.25465	KHVS	4	469.26488
	RHE	3	440.21318	KHGE	4	469.2285
	EDM	3	393.12059	QPAY	4	477.22235

**Table 6:** Identification of peptides in the sixth round of soy sauce-flavored Baijiu.

Sample	Sequence	Length	Mass	Sequence	Length	Mass
The sixth round	RKF	3	449.27505	MVTETLNFEHSNIQVKDFIMG	21	2452.1767
	RRQ	3	458.27136	LPQRHRMVYSLL	12	1511.8395
	YHY	3	481.19613	PLDLTSFVLHEAI	13	1453.7817
	RHV	3	410.239	AGDDKKNRD	9	1017.4839
	RQP	3	399.22302	VSRY	4	523.27545
	YPH	3	415.18557	NVLH	4	481.26488
	FCF	3	415.15658	NEWH	4	584.23431
	RNK	3	416.24957	PLFVN	5	588.32715
	HDY	3	433.15975	WWWYL	5	852.3959
	EDM	3	393.12059			

**Table 7:** Identification of peptides in the seventh round of soy sauce-flavored Baijiu.

Sample	Sequence	Length	Mass	Sequence	Length	Mass
The seventh round	RRQ	3	458.27136	MVTETLNFEHSNIQVKDFIMG	21	2452.1767
	RFL	3	434.26415	PLDLTSFVLHEAI	13	1453.7817
	WAK	3	403.22195	MKVP	4	473.26719
	RFH	3	458.239	PPFTG	5	517.25365
	QKL	3	387.24817	NVLH	4	481.26488
	RHV	3	410.239	VSRY	4	523.27545
	VEE	3	375.16416	FFTQ	4	541.25365
	THF	3	403.18557	LFNP	4	489.25873
	YHY	3	481.19613	NYFV	4	541.25365
	MDF	3	411.14641	NEWH	4	584.23431
	WSW	3	477.20122	KHGE	4	469.2285
	TEY	3	411.16416	RMND	4	534.22203
	MMQ	3	408.15011	LEWE	4	575.25913
	RQD	3	417.1972	QPAY	4	477.22235

## Data Availability

The original contributions presented in the study are included in the article/Supplementary Materials; further inquiries can be directed to the corresponding author.

## Supplementary Materials

The following supporting information can be downloaded at: https://www.mdpi.com/article/10.3390/foods14020287/s1. Table S1. Peptides identified in multiple rounds of soy sauce-flavored Baijiu. Table S2. Molecular docking between the typical peptides and the odorous substances. Figure S1. Identification of peptides in the distillates from various round of soy sauce-flavored Baijiu by LC-MS.
