# Supplementary material for: Identification of Oligopeptides in the Distillates from Various Rounds of Soy Sauce-Flavored Baijiu and Their Effect on the Ester–Acid–Alcohol Profile in Baijiu
Source: Foods. 2025 Jan 16;14(2):287. doi: 10.3390/foods14020287 (PMC11764888; doi:10.3390/foods14020287)
Supplement: Supplementary file 1 [file foods-14-00287-s001.zip › foods-3385573-supplementary.pdf]

Supplementary Tables

Table S1 Peptides identified in multiple rounds of soy sauce-flavored Baijiu

Sequence	Length	Mass	The rounds in which the peptides were identified						
			Round 1	Round 2	Round 3	Round 4	Round 5	Round 6	Round 7
NEWH	4	584.23431	✔		✔			✔	✔
NYFV	4	541.25365	✔		✔				✔
FCF	3	415.15658	✔					✔	
KHVS	4	469.26488	✔	✔		✔	✔		
VEE	3	375.16416			✔		✔		✔
LFNP	4	489.25873	✔	✔					✔
PPNF	4	473.22743	✔	✔					
NMLP	4	473.2308	✔		✔				
EDM	3	393.12059		✔			✔	✔	
HDY	3	433.15975		✔				✔	
RQP	3	399.22302					✔	✔	
TEY	3	411.16416		✔	✔				✔
TRH	3	412.21827	✔		✔				
WAK	3	403.22195	✔		✔				✔
WRD	3	475.21793	✔	✔					
WSW	3	477.20122	✔						✔
WVP	3	400.21106	✔			✔	✔		
YHY	3	481.19613		✔				✔	✔
YPH	3	415.18557	✔					✔	
MDF	3	411.14641	✔	✔					✔
RGEY	4	523.23906				✔	✔		
TKAS	4	405.22235		✔			✔		
TREY	4	567.26528		✔		✔			

VSRY	4	523.27545						✔	✔
RHV	3	410.239			✔			✔	✔
NVLH	4	481.26488	✔				✔	✔	✔
TRLF	4	535.31183	✔	✔	✔				
PLFVN	5	588.32715	✔					✔	
KHGE	4	469.2285	✔	✔		✔	✔		✔
PPFTG	5	517.25365	✔		✔				✔
MKY	3	440.20934	✔	✔					
MKVP	4	473.26719			✔				✔
QPAY	4	477.22235	✔		✔		✔		✔
RMND	4	534.22203	✔						✔
RRQ	3	458.27136		✔	✔		✔	✔	✔
LHR	3	424.25465	✔				✔		
QKL	3	387.24817			✔				✔
RHE	3	440.21318	✔				✔		
RKF	3	449.27505		✔	✔		✔	✔	
RNK	3	416.24957			✔		✔	✔	
RNN	3	402.19753		✔	✔				
PLDLTSFVLHEAI	13	1453.7817			✔			✔	✔
LPLEELPAIMEAI	13	1437.7789		✔	✔				
DLYANTVLSGGTTMYPGIADR	21	2214.0627		✔	✔				
LPQRHRMVYSLL	12	1511.8395					✔	✔	
AGDDKKNRD	9	1017.4839	✔					✔	
MVTETLNFEHSNIQVKDFIMG	21	2452.1767	✔		✔		✔	✔	✔
LLGKLGGL	8	769.50617	✔		✔				
VVNIPVVG	8	795.48544	✔		✔				

Table S2 Molecular docking between the typical peptides and the odorous substances

Peptide	Ligand	Vdw-Hb-Desolv energy (kcal/mol)	Electrostatic energy (kcal/mol)	Torsional energy (kcal/mol)	Binding energy (kcal/mol)	Number of hydrogen bonds	Hydrogen bond distance	Residues formed hydrogen bonds with the ligand
TRH	Ethanol	-1.35	-0.53	0.30	-1.58	2	1.592<br>1.763	Thr(H)<br>Arg(O)
	1-Propanol	-1.69	-0.55	0.60	-1.64	2	1.592<br>1.729	Thr(H)<br>Arg(O)
	1-Butanol	-2.01	-0.57	0.89	-1.68	2	1.581<br>1.777	Thr(H)<br>Arg(O)
	1-Hexanol	-2.75	-0.45	1.49	-1.70	2	1.750<br>1.713	Thr(H)<br>Arg(O)
	Acetate acid	-0.93	-2.05	0.30	-2.69	1	1.416	Thr(H)
	Butanoate acid	-1.74	-1.92	0.89	-2.77	1	1.448	Thr(H)
	Hexanoate acid	-2.32	-1.97	1.49	-2.80	1	1.491	Thr(H)
	Lactate acid	-2.01	-2.01	0.89	-3.13	2	1.431<br>1.905	Thr(H)<br>Arg(O)
	Ethyl acetate	-1.67	-0.51	0.60	-1.59	1	1.615	Thr(H)
	Ethyl butanoate	-2.30	-0.43	1.19	-1.53	1	1.791	Thr(H)
	Ethyl hexanoate	-2.77	-0.46	1.79	-1.43	1	1.728	Thr(H)
	Ethyl lactate	-2.69	-0.43	1.19	-1.93	2	1.655<br>1.763	Thr(H)<br>Arg(O)
YHY	Ethanol	-1.41	-0.31	0.30	-1.42	2	1.910<br>1.982	Tyr(H)<br>Tyr(O)
	1-Propanol	-1.77	-0.27	0.60	-1.45	2	1.992<br>1.925	Tyr(H)<br>Tyr(O)
	1-Butanol	-2.08	-0.28	0.89	-1.46	2	2.003<br>1.830	Tyr(H)<br>Tyr(O)
	1-Hexanol	-2.83	-0.23	1.49	-1.56	1	1.773	Tyr(H)
	Acetate acid	-1.21	-1.43	0.30	-2.35	2	2.214<br>1.749	Tyr(H)<br>His(HN)
	Butanoate acid	-1.80	-1.40	0.89	-2.31	2	2.225<br>1.783	Tyr(H)<br>His(HN)

	Hexanoate acid	-2.19	-1.51	1.49	-2.20	1	1.953	Tyr(H)
	Lactate acid	-1.85	-1.55	0.89	-2.50	2	2.022<br>1.622	Tyr(H)<br>His(O)
	Ethyl acetate	-2.41	-0.18	0.6	-2.00	1	2.158	Tyr(H)
	Ethyl butanoate	-2.50	-0.20	1.19	-1.51	1	1.990	His(HN)
	Ethyl hexanoate	-2.99	-0.24	1.79	-1.44	1	2.016	His(HN)
	Ethyl lactate	-2.75	-0.07	1.19	-1.62	1	2.079	Tyr(H)
RQTQ	Ethanol	-1.52	-0.18	0.30	-1.40	1	1.814	Thr(O)
	1-Propanol	-1.88	-0.11	0.60	-1.39	1	2.068	Thr(O)
	1-Butanol	-2.12	-0.09	0.89	-1.32	1	1.929	Thr(O)
	1-Hexanol	-2.75	-0.05	1.49	-1.13	1	1.999	Thr(O)
	Acetate acid	-1.04	-2.19	0.30	-2.93	0	-	-
	Butanoate acid	-0.95	-2.69	0.89	-2.75	2	2.197<br>1.490	Arg(HN)<br>Arg(H)
	Hexanoate acid	-2.06	-2.04	1.49	-2.61	0	-	-
	Lactate acid	-0.58	-2.82	0.89	-2.51	2	2.075<br>2.464	Arg(HN)<br>Arg(H)
	Ethyl acetate	-1.61	-0.34	0.6	-1.36	1	2.078	Arg(H)
	Ethyl butanoate	-2.42	-0.03	1.19	-1.26	0	-	-
	Ethyl hexanoate	-2.80	-0.12	1.79	-1.14	1	2.069	Gln(H)
	Ethyl lactate	-2.57	-0.02	1.19	-1.39	1	1.995	Gln(O)
PLDLTSFVLHEAI	Ethanol	-2.16	-0.03	0.30	-1.89	1	1.764	Phe(O)
	1-Propanol	-2.63	-0.08	0.60	-2.12	2	2.170<br>1.736	Ala(H)<br>Phe(O)
	1-Butanol	-3.04	-0.09	0.89	-2.23	2	2.088<br>1.782	Ala(H)<br>Phe(O)
	1-Hexanol	-3.74	-0.05	1.49	-2.30	1	1.981	Phe(O)
	Acetate acid	-1.08	-1.31	0.30	-2.10	1	1.484	Pro(H)
	Butanoate acid	-2.80	-0.05	0.89	-1.96	0	-	-
	Hexanoate acid	-3.54	-0.06	1.49	-2.11	0	-	-
	Lactate acid	-2.85	-0.02	0.89	-1.97	1	1.951	Phe(O)
	Ethyl acetate	-2.94	0.03	0.60	-2.31	0	-	-
	Ethyl butanoate	-3.76	0.03	1.19	-2.54	0	-	-
	Ethyl hexanoate	-4.24	0.04	1.79	-2.41	1	2.945	Ser(O)

	Ethyl lactate	-3.82	-0.02	1.19	-2.65	1	2.092	Phe(O)
KHVS	Ethanol	-1.40	-0.27	0.30	-1.38	0	-	-
	1-Propanol	-1.78	-0.26	0.60	-1.45	0	-	-
	1-Butanol	-2.11	-0.22	0.89	-1.43	2	2.124<br>2.019	Lys(H)<br>Lys(O)
	1-Hexanol	-2.75	-0.17	1.49	-1.43	2	2.161<br>1.931	Lys(H)<br>Lys(O)
	Acetate acid	-0.97	-2.44	0.30	-3.12	1	2.181	Lys(H)
	Butanoate acid	-1.74	-2.35	0.89	-3.19	0	-	-
	Hexanoate acid	-2.34	-2.30	1.49	-3.15	0	-	-
	Lactate acid	-1.90	-1.91	0.89	-2.91	2	1.971<br>1.756	Lys(H)<br>Lys(O)
	Ethyl acetate	-1.78	-0.42	0.60	-1.60	1	2.774	Lys(O)
	Ethyl butanoate	-2.53	-0.22	1.19	-1.56	1	2.997	Lys(O)
	Ethyl hexanoate	-2.99	-0.42	1.79	-1.63	1	2.616	Lys(O)
	Ethyl lactate	-2.67	-0.25	1.19	-1.72	1	1.912	Lys(O)
LPQRHRMVYSLL	Ethanol	-2.02	-0.09	0.30	-1.82	1	1.767	Met(O)
	1-Propanol	-2.52	-0.09	0.60	-2.01	1	1.722	Met(O)
	1-Butanol	-2.74	-0.08	0.89	-1.92	1	1.896	Arg(O)
	1-Hexanol	-3.53	-0.05	1.49	-2.08	1	1.986	Val(O)
	Acetate acid	-1.15	-1.59	0.30	-2.44	3	2.013<br>1.797<br>1.709	Arg(H)<br>Arg(HN)<br>Arg(H)
	Butanoate acid	-1.94	-1.59	0.89	-2.63	3	2.048<br>1.808<br>1.716	Arg(H)<br>Arg(HN)<br>Arg(H)
	Hexanoate acid	-2.93	-1.56	1.49	-3.01	3	2.037<br>1.741<br>1.008	Arg(H)<br>Arg(HN)<br>Arg(H)
	Lactate acid	-2.45	-0.94	0.89	-2.49	1	2.144	Arg(O)
	Ethyl acetate	-2.75	-0.01	0.60	-2.16	1	2.221	Arg(O)
	Ethyl butanoate	-3.56	0.00	1.19	-2.37	0	-	-
	Ethyl hexanoate	-4.22	0.03	1.79	-2.40	1	2.816	Met(O)
	Ethyl lactate	-3.54	-0.01	1.19	-2.35	1	1.893	Met(O)

NEWH	Ethanol	-1.35	-0.38	0.30	-1.43	2	2.180<br>2.054	Glu(O)<br>Asn(H)
	1-Propanol	-1.90	-0.18	0.60	-1.48	1	1.802	Glu(O)
	1-Butanol	-2.33	-0.04	0.89	-1.47	1	2.024	Glu(O)
	1-Hexanol	-3.04	-0.13	1.49	-1.68	1	1.913	Glu(O)
	Acetate acid	-0.86	-1.66	0.30	-2.22	0	-	-
	Butanoate acid	-1.76	-1.59	0.89	-2.45	0	-	-
	Hexanoate acid	-2.45	-1.39	1.49	-2.35	1	1.673	Asn(H)
	Lactate acid	1.69	-1.45	0.89	-2.25	2	1.887<br>1.427	Asn(O)<br>Asn(H)
	Ethyl acetate	-1.98	-0.20	0.60	-1.58	1	-	-
	Ethyl butanoate	-2.57	-0.08	1.19	-1.45	1	2.758	
	Ethyl hexanoate	-3.20	-0.06	1.79	-1.47	0	-	-
	Ethyl lactate	-2.88	-0.12	1.19	-1.81	1	1.806	Glu(O)

Supplementary Figure

Figure S1. Identification of peptides in the distillates from various round of soy sauce-flavored Baijiu by LC-MS. (A) Total ion chromatogram; (B) The corresponding second-order mass spectrum of TRH; (C) The corresponding second-order mass spectrum of YHY; (D) The corresponding second-order mass spectrum of RQTQ; (E) The corresponding second-order mass spectrum of PLDLTSFVLHEAI; (F) The corresponding second-order mass spectrum of KHVS; (G) The corresponding second-order mass spectrum of LPQRHRMVYSLL; (H) The corresponding second-order mass spectrum of NEWH.

A

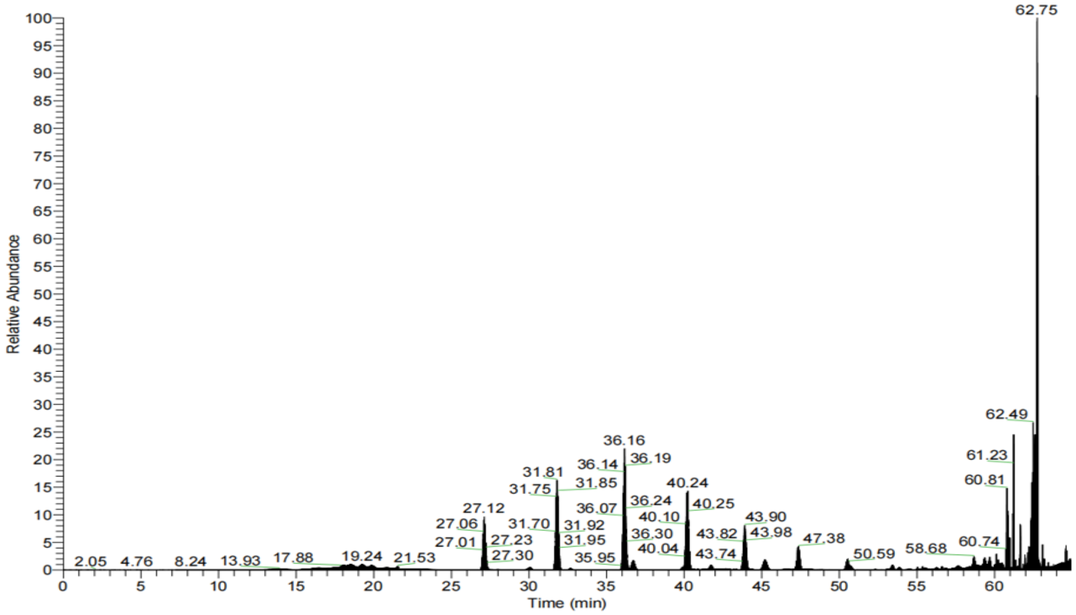

B

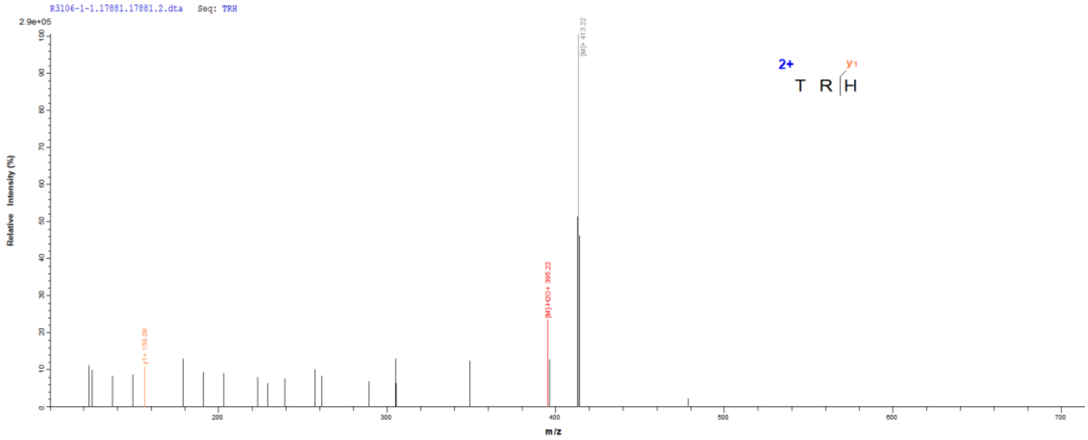

C

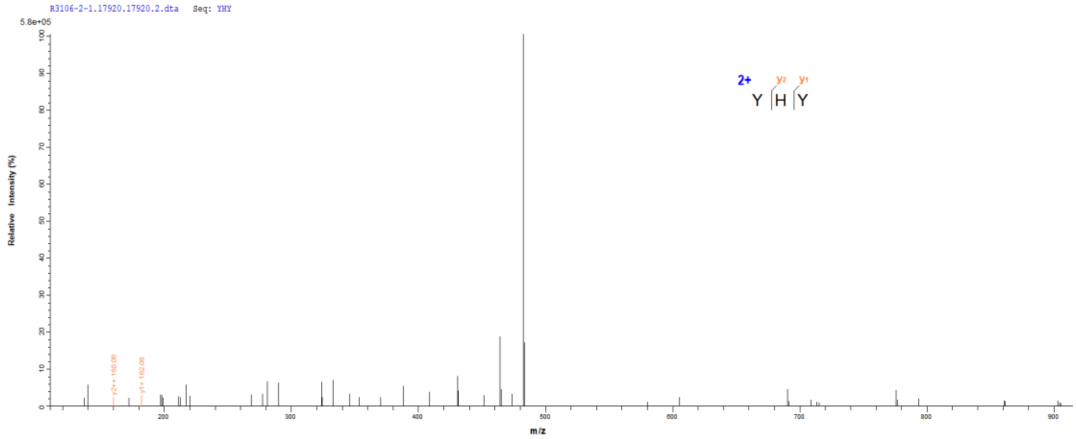

D

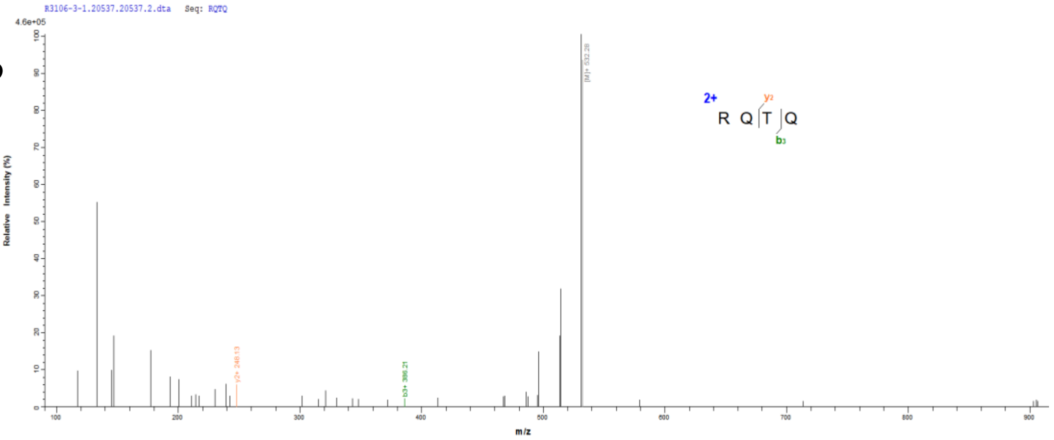
R3106-3-1.20537.20537.2.dta Seq: RQTQ
4.6e+05
Relative Intensity (%)
m/z
2+
R Q T Q

E

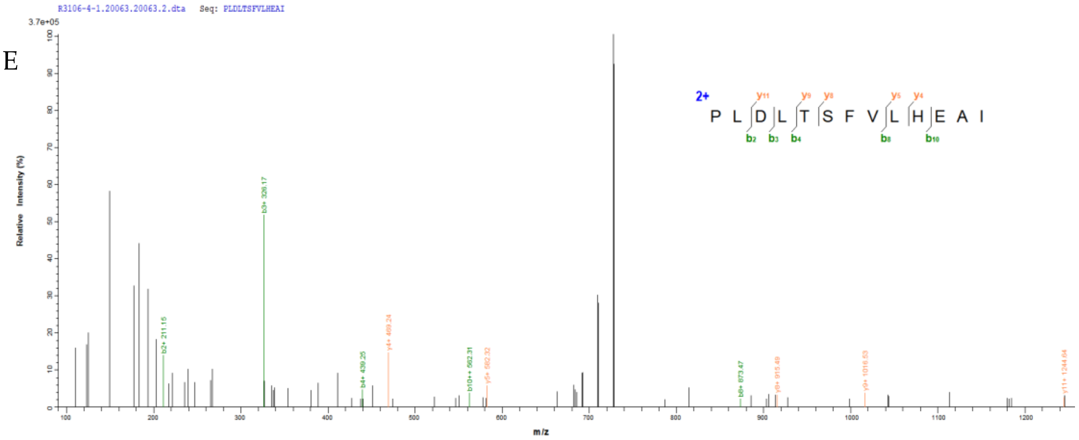
R3106-4-1.20063.20063.2.dta Seq: PLDLTSFVLHEAI
3.7e+05
Relative Intensity (%)
m/z
2+
P L D L T S F V L H E A I

F

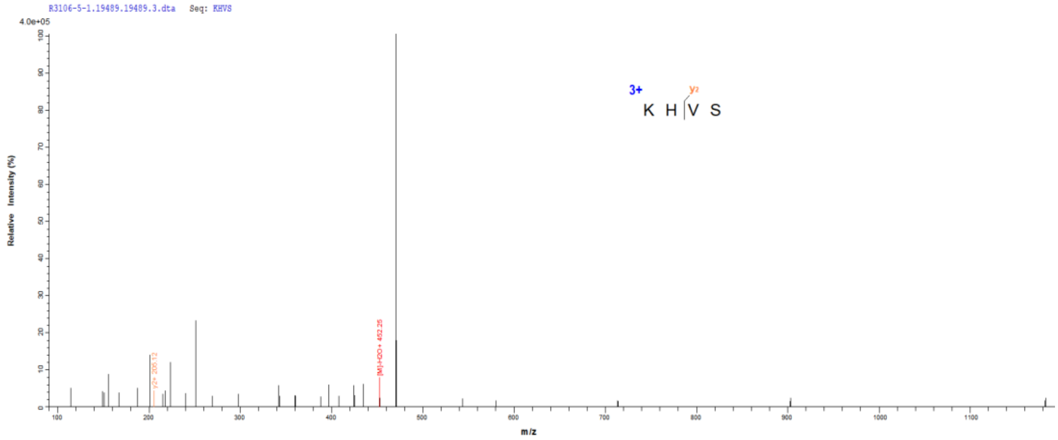
R3106-5-1.19489.19489.3.dta Seq: KHVS
4.0e+05
Relative Intensity (%)
m/z
3+
K H V S

G

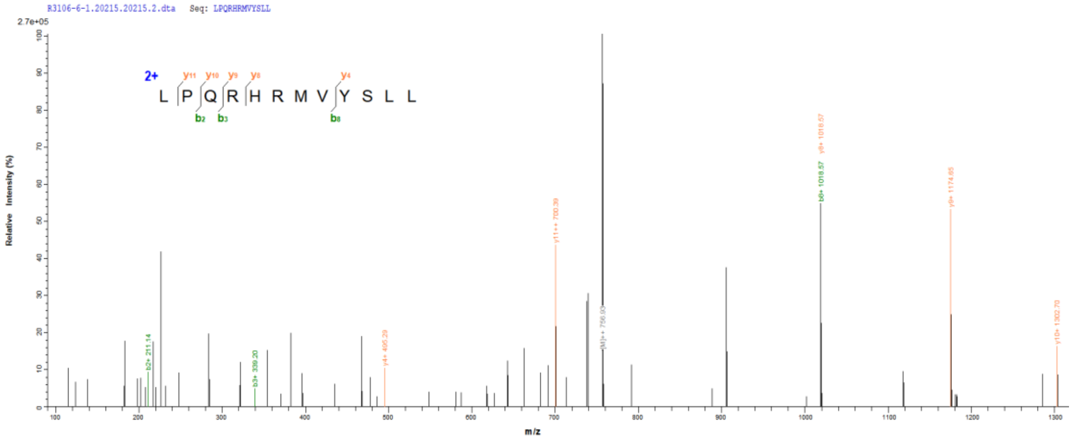
R3106-6-1.20215.20215.2.dta Seq: LPQRHRMVYSLL
2.7e+05
Relative Intensity (%)
m/z
2+
L P Q R H R M V Y S L L

H

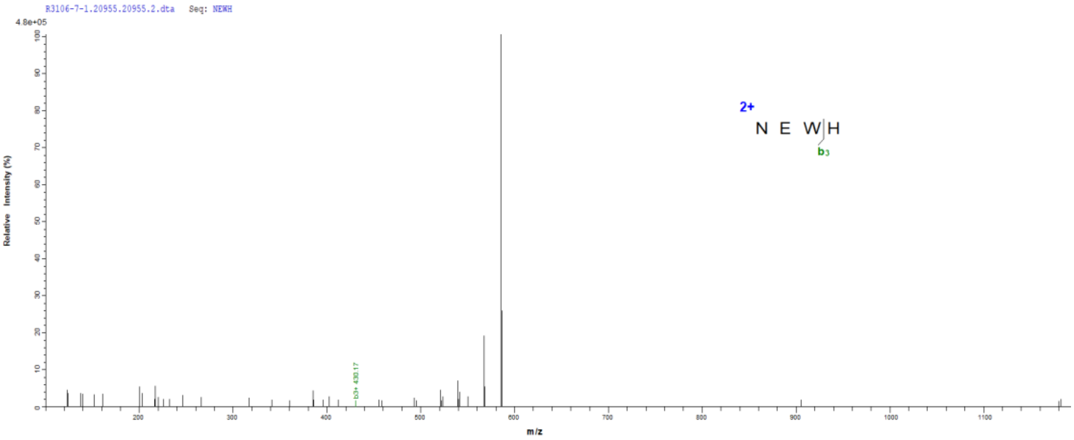
R3106-7-1.20955.20955.2.dta Seq: NEWH
4.8e+05
Relative Intensity (%)
0
10
20
30
40
50
60
70
80
90
100
b3+ 430.17
200
300
400
500
600
700
800
900
1000
1100
m/z
2+
N E W H
b3
